# Four-week administration of an energy and protein dense oral nutritional supplement improves micronutrient concentrations but does not completely correct deficiencies in institutionalized malnourished older adults

**DOI:** 10.3389/fnut.2023.1249936

**Published:** 2023-09-27

**Authors:** Manuel Sanchez, Pauline Courtois-Amiot, Audrey Capdepon, Nathalie Neveux, Julien Gautry, Béatrice Dorigny, Ludovic Brossault, Olivier Bouillanne, Christian Aussel, Agathe Raynaud-Simon, Luc Cynober

**Affiliations:** ^1^Department of Geriatrics, APHP Bichat Hospital, Paris, France; ^2^Faculty of Medicine, Paris Cité University, Paris, France; ^3^Nestlé Health Science, Issy-les-Moulineaux, France; ^4^Research Unit in Pharmacology URP4466, Faculty of Pharmacy, Paris Cité University, Paris, France; ^5^Clinical Chemistry Laboratory, APHP Cochin Hospital, Paris, France; ^6^Soladis Group, Lyon, France; ^7^Department of Geriatrics, APHP Emile Roux Hospital, Limeil-Brévannes, France; ^8^Clinical Chemistry Laboratory, APHP Saint-Antoine Hospital, Paris, France

**Keywords:** magnesium, malnutrition, micronutrient, nursing home, older adults, selenium, vitamin, zinc

## Abstract

**Introduction:**

Poor food intake is common among elderly living in nursing homes, leading to micronutrient deficiency (MD). There are no recommendations for the management of MD in malnourished older adults.

**Methods:**

We conducted a single arm, open-label, multicenter interventional study in institutionalized malnourished older adults to describe the effect of a 4-week daily energy and protein dense oral nutritional supplementation (ONS, 600 kcal, 30 g protein per unit) containing 50% of the recommended daily micronutrient intake on micronutrient status. Plasma concentrations of vitamins (A, B9, B12, C, E), magnesium (Mg), selenium (Se) and zinc (Zn), and erythrocyte vitamin B9 were measured at baseline and after 4 weeks.

**Results:**

Forty-six participants completed the study (age 87.4 ± 6.6). At baseline, the most frequent MD were Se (48%), Zn (35%), Mg (24%) and vitamin C (24%). Plasma concentrations of vitamins B9, B12, C and E, Mg, Se and Zn significantly increased and the proportion of subjects with at least one MD decreased (*p* = 0.006). However, after 4 weeks, 40% of subjects still had at least one MD.

**Discussion:**

ONS consumption improved micronutrient status but did not correct MD in all participants. Our data suggest that the prescription of vitamin, mineral and trace element supplementation should be considered in institutionalized malnourished older adults in addition to high energy and high protein ONS.

## 1. Introduction

Poor food intake is still common among older adults living in nursing homes, leading to protein-energy malnutrition and micronutrient deficiency (1, 2). Protein-energy malnutrition has been extensively addressed, leading to guidelines and recommendations for the nutritional care and oral nutritional supplements (ONS) of older adults, including those living in nursing homes (3, 4). In contrast, the deficiency in micronutrients due to low food intake in older adults living in nursing homes has received little attention.

In nursing homes, a high prevalence of inadequate intake of many vitamins, trace elements and minerals (retinol, vitamins B2, B6, B9, B12, C, E, potassium, calcium, magnesium, copper and zinc) have been reported in several countries (5–11). Residents consuming pureed food seem to be particularly at risk of low micronutrient intake (11, 12). Biological measurements suggest that the most important deficiencies affecting residents are zinc, selenium, vitamins B1, B6, B9, B12 and C (10, 13–15). These deficiencies may lead to various neurological, psychiatric, hematological, and skin disorders affecting quality of life and survival. In addition, numerous micronutrients are essential for the proper functioning of the immune system (16). Especially, zinc deficiency leads to a dysregulation in the adaptive immunity that can result in an increased production of pro-inflammatory cytokines (contributing to inflammaging), as well as a decreased response to vaccination (17). Furthermore, optimal status for zinc and vitamin C are mandatory for the healing of chronic wounds such as pressure sores (18). Magnesium has an important role in maintaining muscle function in aging animals and human. In aged mice, magnesium supplementation promotes muscle regeneration and maintains muscle mass and strength by activating the mammalian target of rapamycin signaling (19). In humans, magnesium intake is inversely associated with sarcopenia (20). Selenium deficiency is also frequent in Western older populations (21). Selenium has antioxidant properties, an active role in endothelial function, and could act as one of key actors of cardiovascular prevention (22). Older adults are especially at risk for vitamin B12 deficiency because of frequently inadequate animal-source food intake (23). Vitamin B12 deficiency is suspected to promote cognitive decline. Lastly, most symptoms of vitamin B9 deficiency overlap with those of vitamin B12 deficiency, including depression and cognitive disorders (24). However, despite potentially serious consequences on the quality of aging, micronutrient deficiencies are difficult to diagnose because clinical symptoms may be unspecific or attributable to aging or comorbidities. In addition, micronutrient plasma measurements are not routinely performed in malnourished subjects. Practitioners may be at loss as how to cope with micronutrient deficiencies as there are no guidelines regarding multi-micronutrient supplementation in malnourished older adults.

Multi-micronutrient supplementation increases micronutrient plasma concentrations in malnourished older adults (13–15, 25, 26), but are not routinely prescribed in nursing home residents. Energy and protein dense ONS that are recommended for malnourished older adults do contain micronutrients, as regulated by country guidelines (27), but it is not clear if the micronutrient content of ONS is high enough to improve or correct micronutrient status. We found three studies describing the change in plasma micronutrient status in nursing home residents after ONS prescription (with no additional micronutrient supplementation) (15, 26, 28). The changes in plasma concentrations of selected micronutrients were measured before and after 10–24 weeks of ONS providing 272–500 kcal, 8.5–15 g of protein, and vitamins, minerals and trace elements in varying quantities. Fiatarone Singh et al. (28) reported no significant change in folates, iron, vitamin D and E. In the second study (26), plasma concentrations of B6, B9 and B12 improved significantly, but only that of B6 and B9 reached the normal range for all residents after 12 weeks. In the third study (15), plasma concentrations of vitamin D, B9 and B12 improved significantly but only B9 levels reached the normal range for all residents. Thus, the micronutrient status of malnourished older adults living in nursing homes receiving ONS needs to be further explored.

The ONS Renutryl Booster (Nestlé Health Science) was especially formulated for older adults, is rich in energy and protein and the composition includes 50% of micronutrient French RDA for older adults (29). We aimed to describe the effect of 1-month energy and protein dense oral nutritional supplementation of this ONS on micronutrient status (magnesium, selenium, zinc, vitamins A, B9, B12, C, and E) in malnourished older adults living in nursing homes.

## 2. Materials and methods

### 2.1. Study design, selection of participants and ethics

Between 2012 and 2014, we conducted a single arm, open-label, multicenter interventional study in nursing homes and long-term care facilities. Nineteen facilities agreed to participate in the study: five were investor-owned for-profit facilities, seven were non-profit institutions, and seven were public facilities. During the opening visits of the centers, the medical and paramedical staff of each center were informed to the study protocol and trained for the assessment of anthropometric measures, body composition, physical performances and for the quantification of ONS consumption. Inclusion criteria were age ≥70 years and moderate malnutrition. Following French Health Authority recommendations at the time of the study (30), moderate malnutrition was defined by the presence of at least one of the following criteria: body weight loss ≥5% in 1 month or ≥10% in 6 months, or body mass index (BMI) <21, plasma albumin <35 g/L or Mini Nutritional Assessment—Short Form (MNA-SF) ≤7. Non-inclusion criteria were (a) severe malnutrition, defined by the presence of at least one of the following criteria: body weight loss ≥10% in 1 month or ≥15% in 6 months, BMI <18, plasma albumin <30 g/L; (b) no oral feeding; (c) life expectancy <6 months; (d) inflammatory syndrome with C-reactive protein (CRP) >30 mg/L; (e) stage IV in heart failure according to the New-York Heart Association; (f) chronic kidney disease with estimated glomerular filtration rate <30 ml/min; (g) pulmonary disease requiring long-term oxygen therapy; (h) severe dementia [Mini Mental State Examination score (MMSE) <10] (31); (i) inability to walk 6 m alone with or without a walk-assisting device; (j) treatment within the last 3 months with systemic corticosteroid therapy; (k) enteral or parenteral nutrition, amino acid supplementation, or supplementation within the last 3 months with any ONS or micronutrients (magnesium, zinc, selenium, vitamins A, B9, B12, E, or C); and (l) intolerance to ONS. All study participants (or their legal guardian) were given oral and written information about the study protocol and signed an informed consent prior to inclusion. The study protocol was approved by the Institutional Review Board *Comité de Protection des Personnes Ile-de-France*—*Paris VI* as n°63-12, and was registered in the French National Database ID-RCB as 2012-A00740-43.

### 2.2. Oral nutritional supplementation

The ONS Renutryl Booster^®^ (Nestlé Health Science) used in this study is a 300 ml liquid milk ONS with a high density in energy (2 kcal/ml, 600 kcal for 300 ml) and protein (30 g for 300 ml). The micronutrient composition of this ONS is given in Table 1. The micronutrient composition is in accordance with the European policies (27, 32). Each participant was asked to consume one bottle of the ONS daily for 28 days. Participants could choose among different flavors (vanilla, strawberry, caramel, coffee, carrot cream). They could choose a different flavor each day, and consume the bottle all at once or spread throughout the day. ONS intake was assessed by weighing the bottle the day following delivery. Caregivers from each nursing home were trained to weigh the bottles and complete the compliance chart. Compliance was expressed in percentage (volume weighed/300) per day and expressed as median [Q1–Q3] for the 4 weeks' duration of the study.

**Table 1:** Oral nutritional supplement composition (per unit of 300 ml).

		**Sweet flavor**	**Salty flavor**
Energy	Kcal	600	600
Protein	g	30	30
	%	20%	20%
Carbohydrate	g	72	72
	%	48.5	48.5
Sucrose	g	21	21
Lactose	g	< 1.5	< 1.5
Lipid	g	21	21
	%	31.5	31.5
**Minerals and trace elements**			
Sodium	mg	285	584
Potassium	mg	720	720
Calcium	mg	687	687
Phosphorus	mg	459	459
Magnesium	mg	150	150
Chlorides	mg	255	564
Iron	mg	5.1	5.1
Zinc	mg	7.5	7.5
Copper	μg	750	750
Manganese	μg	498	498
Fluorine	mg	1.2	1.2
Chrome	μg	63	63
Molybdenum	μg	21	21
Selenium	μg	39	39
Iodine	μg	75	75
**Vitamins**			
A	μg	351	351
D	μg	5.1	5.1
E	mg	10.2	10.2
K	μg	36	36
C	mg	60	60
B1	mg	0.6	0.6
B2	mg	0.8	0.8
B5	mg	2.5	2.5
B6	mg	1.11	1.11
B12	μg	2.4	2.4
Niacin	mg	13.5	13.5
Folic acid	μg	201	201
Biotin	μg	30	30
Osmolarity	mOsmol/L	580	530

### 2.3. Clinical data

Clinical data was collected on day 1 and day 29 by a trained investigator. Data included age, gender, comorbidities, cognitive performance (MMSE) and disability for activities of daily living (ADL) (33). The nursing home medical record was used to record comorbidities, including hypertension, diabetes mellitus, cancer, cardiovascular, psychiatric, neurological, and osteoarticular disorders. The number of current medications was recorded as an indicator of comorbidity. Moreover, in order to consider potential drug-nutrient interactions, we determined the proportion of participants taking drugs known to interact with micronutrients availability (proton pump inhibitors, statins, metformin, loop and thiazide diuretics) at baseline (34). We also described what drugs were interrupted or newly prescribed during the participation in the study. Nutritional status was assessed by MNA-SF, BMI and recent weight loss. Participants with severe or moderate decline in food intake in the past 3 months were identified using the first item of the MNA-SF. Physical performance was assessed using the Timed Up and Go (TUG) test (35). Body composition was measured using bioelectrical impedance analysis (Analycor 4/5W analyzer, Spengler, Cachan, France); fat mass and fat free mass were measured in kg at day 1 and day 29.

### 2.4. Micronutrient assays and laboratory tests

Nursing home nurses drew blood samples of each participant in the morning after a fasting night on day 1 (before the first ONS intake) and on day 29 (the day after the last ONS intake). Five test tubes were collected: (a) two 5-ml dry tubes for albumin, transthyretin, total cholesterol, triglycerides, vitamins A, E, B9, and B12 determinations, (b) two 5-ml heparinized tubes for CRP, magnesium, vitamin C, selenium, and zinc determinations, and (c) one 5-ml ethylenediaminetetraacetic acid (EDTA) tube for erythrocyte folate determination. The test tubes were transported in an isothermal bag at four degrees Celsius to the Clinical Chemistry Laboratory of the Cochin University Hospital within 3 h of collection. For geographically distant centers, the pre-analytical phase was performed in a local laboratory, then the tubes were sent at −80°C to the Biological Chemistry Laboratory of Cochin University Hospital where all tests were performed except for Zn and Se which were analyzed by Cerba Laboratory (ZI Des Bethunes 11 r Equerre, 95310 Saint Ouen l'Aumône, France). Vitamins A, E, and C were measured by reversed-phase high-performance liquid chromatography with fluorometry (vitamins A and E) (36) or ultraviolet (vitamin C) (37) detection. Vitamin E concentrations were corrected for cholesterol and triglyceride levels. Vitamin B9 (serum and erythrocytes) and B12 were assessed by electrogenerated chemiluminescence (38). Magnesium was assessed by colorimetry and the zinc and selenium by Atomic absorption spectrometry (39). To define micronutrient deficiency we used thresholds proposed in the literature (40–45). Moreover, the analytical characteristics of micronutrient assays (including the normal ranges for the laboratory) are shown in Supplementary Table 1.

### 2.5. Statistical analyses

Continuous variables were expressed as mean and standard deviation (SD) or median with first (Q1) and third (Q3) quartile. Categorical variables were expressed as number and percentage. The clinical variables were compared between values at day 1 and at day 29 using paired Student *t*-test or Wilcoxon test for paired samples for continuous variables or McNemar test for categorical variables. The primary aim of the study was first to determine the efficacy of ONS consumption for increasing the plasma and erythrocyte concentrations of all measured micronutrients globally, and not for a single one in particular. For this purpose, we used the O'Brien method (46), designed to test the efficacy of interventional clinical trials on multiple endpoints. According to this method, we built a composite score reflecting global micronutrient status, after checking on multi-linearity and correlation between variables (plasma concentration of magnesium, selenium, zinc, vitamins A, B9, B12, C, and E). Multi-normality of the micronutrient plasma concentrations was rejected (Kolmogorov-Smirnov test), and none of the variables were strongly or inversely correlated to others. Thus, the application conditions were met to use the non-parametric O'Brien Rank sum test for paired samples, in order to compare the composite score between day 1 and day 29. This test takes into account the alpha risk inflation in case of multiple comparisons. Then, we compared the change of each micronutrient concentration between day 1 and day 29 using the paired Student *t*-test. In addition, we compared the proportion of participants with at least one micronutrient deficiency between day 1 and day 29 using the McNemar test. Lastly, for each micronutrient we also gave the proportion of participants presenting a deficiency at day 1 and at day 29 and performed the McNemar test to compare the proportions between day 1 and day 29 when the application condition was respected (at least five discordant pairs in the 2 × 2 contingency table). Considering a power of 0.8 and an alpha risk of 0.05, the minimum sample size was estimated at 36 participants. Considering the risks of missing data and protocol deviations, we aimed to include a minimum of 55 participants. The analysis used the SAS 9.4. software (SAS Institute, Cary, NC, USA). A *p*-value < 0.05 was considered for significance.

## 3. Results

**Figure 1:**
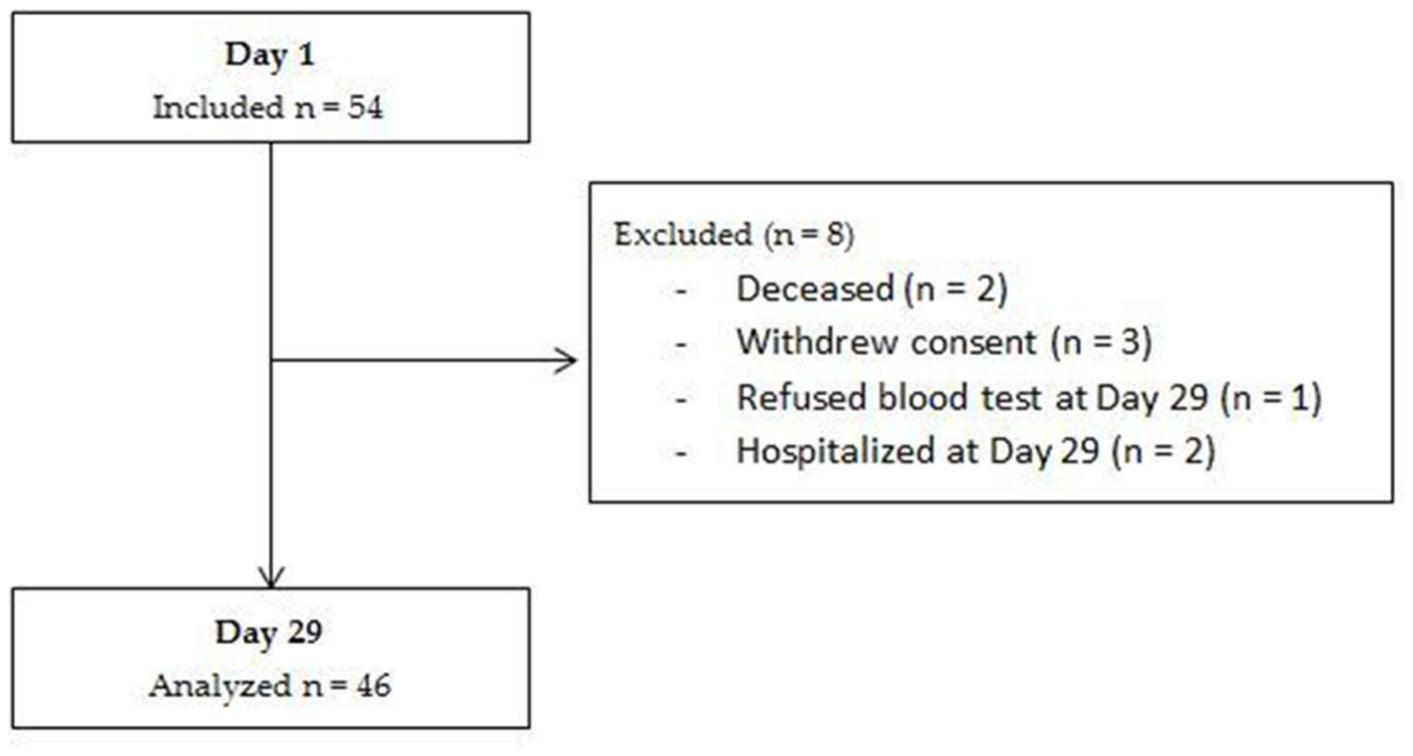
Flow-chart of the subjects included and analyzed in the study.

We included 54 participants recruited in 12 of the 19 facilities that had agreed to participate in the study. Inclusions were not simultaneous in the different institutions, with dates ranging from November 2012 to July 2014. Among the included participants, 46 completed the study and were included in the analyses (Figure 1). Participant's characteristics at baseline are presented in Table 2. The mean age was 87 years, and 76% were women. Half of the participants had three or more chronic conditions, mainly cardiovascular, psychiatric, neurological and osteoarticular disorders. Consequently, polypharmacy was frequent with an average of six medications per patient. Ten (22%) participants were treated with proton pump inhibitors, nine (20%) with statins, nine (20%) with loop diuretics, four (9%) with thiazide diuretic, and two (4%) with metformin at baseline. None of these medications were interrupted during the 4-week intervention and none were newly prescribed. All participants were moderately malnourished, in accordance with inclusion criteria, as showed by rates of low BMI, albuminemia, MNA-SF and by weight loss during the last 3 months. Participants consumed a median of 85% (Q1–Q3: 67%−94%) of the ONS volume given during the 4 weeks.

**Table 2:** Baseline characteristics of the population (*n* = 46).

	**Mean ±SD or *n* (%)**
Women	35 (76%)
Age (year)	87.4 ± 6.6
Activities of daily living (ADL/6)	4.5 ± 1.2
Mini mental state examination (/30)	19.8 ± 5.7
**Comorbidities**	
Psychiatric disorders	19 (41%)
Cardiovascular disorders	16 (35%)
Neurological disorders	11 (24%)
Osteoarticular disorders	11 (24%)
Cancer	7 (15%)
Diabetes	4 (9%)
Polypharmacy (number of drugs)	6.3 ± 2.9
**Proportion of participant treated with**	
Proton pump inhibitors	10 (22%)
Statins	9 (20%)
Loop diuretic	9 (20%)
Thiazide diuretic	4 (9%)
Metformin	2 (4%)
**Nutritional status**	
MNA-SF (/14)	8.2 ± 2.0
MNA-SF ≤ 7	14 (30%)
BMI	21.6 ± 3.1
BMI < 21	29 (63%)
Weight loss	6 (13%)
≥5% in 1 month	5 (11%)
≥10% in 6 months	1 (2%)
Albuminemia (g/L)	36.8 ± 4.0
Albuminemia < 35 g/L	15 (33%)
Transthryretinemia (mg/L)	224 ± 61

BMI, body mass index; MNA-SF, Mini Nutritional Assessment—Short Form.

Plasma concentration of all analyzed micronutrients and erythrocyte level of B9 increased significantly from Day 1 to Day 29, with the exception of vitamin A, for which the concentration increased, but not with statistically significance (Table 3). The composite score showed an improvement in global micronutrient status (*p* < 0.0001). Transthyretinemia also increased significantly (*p* = 0.001) between day 1 and day 29, while the number of participants with low food intake decreased (*p* = 0.006) and participants put on a mean of 1.1 kg (*p* = 0.0001). There were no significant changes in body composition or in physical performance assessed by TUG (Table 3).

**Table 3:** Change in micronutrients concentrations and clinical parameters in 4 weeks (*n* = 46).

	**Day 0**	**Day 29**	***p*-value**
**Micronutrient (thresholds for deficiency)** ^*^			
Magnesium [ < 0.76 mmol/L (41)]	0.80 ± 0.11	0.85 ± 0.09	< 0.001
Selenium [ < 0.75 μmol/L (40)]	0.78 ± 0.17	1.07 ± 0.24	< 0.001
Zinc [ < 10.7 μmol/L in female (45) or < 11.3 μmol/L in male (45)]	11.6 ± 2.1	12.2 ± 2.2	0.016
Vitamin A [ < 0.7 μmol/L (44)]	2.02 ± 0.60	2.16 ± 0.82	0.180
Vitamin E [ < 2.22 μmol/mmol (43)]	5.1 ± 1.1	5.5 ± 1.2	< 0.001
Vitamin C [ < 4.05 mg/L (40)]	8.7 ± 4.7	10.3 ± 3.9	0.005
Vitamin B9 serum [ < 4.0 μg/L (42)]	5.7 ± 2.0	9.0 ± 2.8	< 0.001
Vitamin B9 erythrocyte [ < 151 μg/L (42)]	606 ± 184	714 ± 778	< 0.001
Vitamin B12 [ < 203 ng/L (42)]	388 ± 193	410 ± 223	0.032
Residents with at least one low micronutrient level	38 (86)	17 (40)	< 0.001
Micronutrient deficiencies (number)	1.7 ± 1.1	0.5 ± 0.7	< 0.001
Composite score	384 ± 14	531 ± 15	< 0.001
**Other biological data**			
Albumin (g/L)	36.8 ± 4.0	36.4 ± 4.1	0.547
Transthyretin (mg/L)	224 ± 61	243 ± 65	0.001
**Clinical parameters**			
Moderate or severe decline in food intake	34 (74)	23 (50)	0.006
Body weight (kg)	55.9 ± 10.2	57.0 ± 10.3	< 0.001
Fat mass (kg)^**^	19.2 ± 5.8	18.8 ± 5.6	0.308
Fat free mass (kg)^**^	33.9 ± 6.8	34.4 ± 6.6	0.466
Timed up and go test (seconds)	19.7 ± 6.8	19.8 ± 6.5	0.757

Data are expressed as mean ± SD or number (percentage). The composite score reflects global micronutrient status (log-transformed plasma concentration of magnesium, selenium, zinc, vitamins A, B9, B12, C and E). A higher score reflects a better micronutrient status.

^*^Threshold chosen to define micronutrient insufficiency based on literature (reference).

^**^Missing data for body composition in five participants.

**Figure 2:**
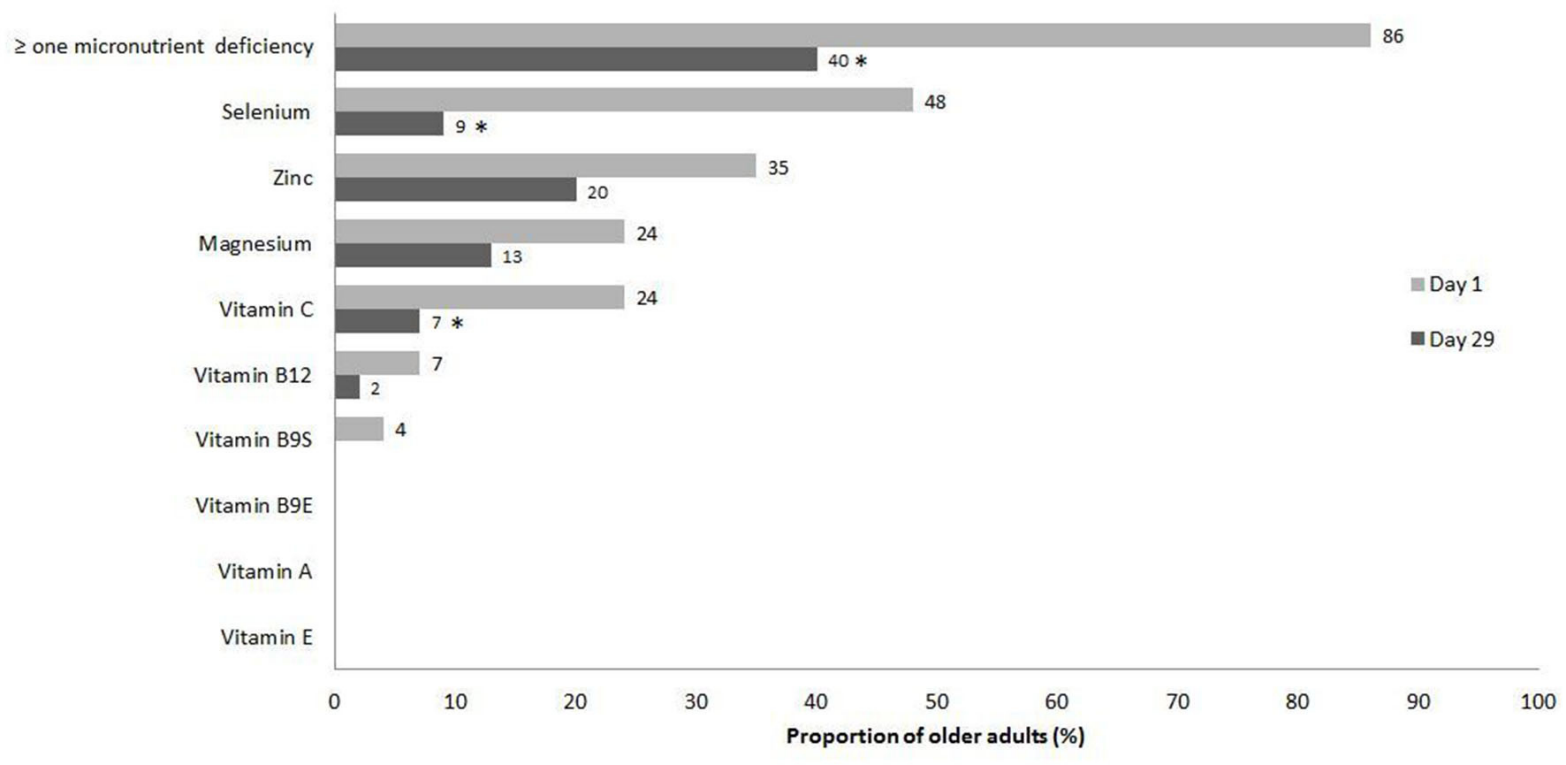
Proportion of participants with micronutrients deficiencies at day1 and day 29. Barres indicates the proportion of older adults with deficiency at day 1 and day 29. ^*^Statistical significance (*p* < 0.05) for the comparison between day 1 and day 29 with the McNemar test.

Figure 2 shows the proportion of participants with micronutrients deficiencies at day 1 and day 29. The most frequently observed deficiencies at baseline were selenium (48%), zinc (35%), magnesium (24%) and vitamin C (24%). The proportion of participants that had at least one micronutrient deficiency was reduced from 86 to 40% (*p* = <0.001) after 4 weeks (Figure 2).

## 4. Discussion

A first step toward improving management of micronutrient deficiencies in malnourished older adults is to determine if energy and protein dense ONS prescription restores or, at least, improves micronutrient status. We show, in the present study, that multi micronutrient deficiency affected a large majority of nursing home residents and that a 4-week daily intake of an energy and protein dense ONS containing micronutrients significantly improved micronutrient status. However, micronutrient plasma levels did not reach normal levels for all participants within the 4 weeks of the study.

In our study, the most frequently observed deficiencies at baseline were selenium (48%), zinc (35%) and vitamin C (24%), which have antioxidant proprieties. Deficiencies in other antioxidant micronutrients were less prevalent, e.g., 13% for vitamin A and none for vitamin E. In comparison, the prevalence of antioxidant micronutrient deficiencies in other populations of nursing home residents were 38%−68% for selenium, 19%−72% for zinc and 35%−75% for vitamin C (25, 47–49). In another study, baseline zinc plasma concentrations were similar to what we observed (50), but the prevalence of the deficiency was not mentioned. Beta-carotene was low in 15% of residents in another study and there was no deficiency in tocopherol (25), which is very similar to what we observed. Overall, and even though data from the literature are scarce, our results confirm a high prevalence of selenium, zinc and vitamin C deficiency at baseline in nursing home residents.

In our study, ONS intake reduced substantially the prevalence of selenium, zinc, and vitamin C deficiencies, which may have a beneficial effect on the health of this population. In a randomized study (25, 51), older institutionalized adults received daily, for 1 year, a supplementation of either vitamins (beta-carotene, vitamin C, and vitamin E), trace elements (zinc and selenium), both or a placebo. It is important to mention that the doses of antioxidant vitamins and trace elements were within physiological range but higher than those in standard high energy high protein ONS (27, 32). Authors report an increase in the mean plasma levels of the micronutrients and an improvement in antioxidant defense and immune response (52). Micronutrient status (especially zinc and vitamin C), also has an important role in the healing of chronic wounds such as pressure sores (18, 48). The prevalence of pressure ulcers varies from 3 to 32% in nursing homes (53). An ONS enriched in zinc, selenium, vitamin C and E and arginine lead to faster reduction in pressure ulcer area than an isoenergetic and isonitrogenous ONS (54). The design of that study did not allow to differentiate the effect of micronutrients from that of arginine on wound healing, but it may be hypothesized that all nutrients concurred to faster healing (18). Lastly, very low vitamin C plasma concentrations will lead to clinical symptoms of scurvy (55), which would in turn impair the residents' ability to eat correctly.

Magnesium deficiency was observed in 24% of residents in our study, and the prevalence was reduced almost by half after 4 weeks ONS intake. Plasma magnesium has seldom been measured in nursing homes, but was reported to be low in 33% of residents (48). In addition to poor food intake, many conditions were liable to lead to hypomagnesemia, including chronic renal failure and poorly-controlled diabetes, and medication such as loop diuretics and proton pomp inhibitors (56), all of which are highly prevalent in the nursing home setting. Indeed, our data showed that more than 20% of the residents were treated with loop diuretics and 22% with proton pump inhibitors. Chronic hypomagnesemia may be implicated in the pathogenesis of various metabolic disorders such as obesity, insulin resistance, hypertension or low-grade inflammation (56). Magnesium supplementation may thus reduce the cardiovascular risk affecting largely institutionalized older adults. In the short term, magnesium supplementation may reduce the risk of atrial fibrillation (57) that affects 7.5%−17% of nursing home residents (58) and reduce blood pressure (59), that can help reduce the risk of cardiovascular and cerebrovascular events. Selenium, as well as zinc deficiencies are associated with the prevalence and prognosis of heart failure (60–62). Neuropsychiatric disorders are also highly prevalent in nursing homes. Deficiencies in B vitamins were associated with an higher risk of depression, whereas B vitamin-fortified foods consumption was associated with a lower risk (63). Observational studies suggest that the association between malnutrition and depression may be partly explained by multiple micronutrient deficiencies (64). The role of B vitamins in cognition is also debated. Higher concentrations in vitamin B12 and folates are associated with better cognitive status in transversal studies, but not with a reduction of cognitive decline in longitudinal studies (65). Results from intervention trials are heterogeneous, thereby making it difficult to conclude on the efficacy of vitamin B supplementation to prevent cognitive decline (66).

ONS are usually prescribed for more than 1 month and it may be hypothesized that the ONS we tested would result in the correction of all deficiencies if prescribed for a longer duration. However, previously published studies that involved ONS prescription up to 24 weeks also did not completely correct micronutrient status (15, 26, 28). We add to these studies by using an ONS that was specially formulated for older adults, with 50% of RDA for older adults (29). In comparison, previously mentioned studies used ONS with either one third of RDA (28), 25%−175% of RDA with enhanced amounts of antioxidants (15), or no reference to RDA (26). In contrast to the composition of ONS used in other studies, we used a richer ONS (600 kcal and 30 g of protein per unit), a composition that reaches or exceeds both energy and protein levels that are recommended for ONS prescription in malnourished older adults: at least 400 kcal and 30 g of protein/day provided by ONS in addition to regular meals (3).

In our study, the improvement in micronutrient status was favored by a notably high compliance to ONS (88%), as compared to previously reported compliance to ONS (67, 68). This high compliance to the ONS was associated with a significant weight gain in only 4 weeks. In the same time, the proportion of residents with “moderate to severe decline in food intake” significantly decreased. This adds to previously reported data suggesting that ONS consumption has little effect on usual food intake and thus increases total energy intake (3, 67). Lastly, no change in body composition and physical performance (Timed Up and Go test) was observed. This may be partly explained by the short duration of the study. However, our study protocol included no physical activity, and thus had little chance of improving muscle mass and function.

In the present study, non-inclusion criteria included chronic kidney disease with estimated glomerular filtration rate <30 ml/min. Bearing this in mind, the ONS was given to all other residents regardless of renal function. On one hand, high protein intake may contribute to accelerate renal failure in patients with chronic kidney disease on the long term. On the other hand, low protein intake may worsen muscle wasting in malnourished subjects, with short-term consequences. Thus, protein restriction is only recommended (69) in “metabolically stable” patients (i.e., absence of any active inflammatory or infectious diseases, no hospitalization within 2 weeks, absence of poorly controlled diabetes and consumptive diseases such as cancer, absence of antibiotic or immunosuppressive medications, and absence of significant short-term loss of body weight).” In addition, oral protein-energy supplementation is recommended in adults with CKD 3–5D (2D) at risk of or with protein-energy wasting, “a minimum of a 3-month trial of oral nutritional supplements to improve nutritional status if dietary counseling alone does not achieve sufficient energy and protein intake to meet nutritional requirements” (69).

Our study has limitations. First, plasma measurements of vitamins and minerals may not reflect perfectly their whole body status. Second, dietary intake of micronutrients was not assessed: this constitutes a potential confounding factor to interpret the results. Also, in this real-life study, the quality, quantity and timing of the regular meals and the rate of ONS consumption (either all at once or spread throughout the day), which were not recorded, may have had an impact on micronutrient absorption and plasma concentration changes. Third, residents could choose each day to consume either a salty or a sweet flavored ONS, but this choice was not rated. The salty ONS contains more sodium and chloride (Table 1), bringing salt (NaCl) intake from 0.7 g in the sweet flavored ONS to 1.5 g in the salty ONS. Although a higher intake of sodium may favor excretion of calcium in the renal proximal tubule (70), sodium and chloride intake is not known to change micronutrient absorption and metabolism (71). Lastly, we did not assess the clinical consequences of micronutrient deficiencies. This point deserves further studies including a larger population. However, very few clinical interventional studies concerning micronutrients in older adults living in nursing homes have been reported, and most of them have been published more than 20 years ago (13, 25, 26, 28). We add to these studies by reporting baseline and changes in plasma concentrations of eight vitamins and trace elements in response to consumption of a high energy and high protein ONS.

In conclusion, we showed that an ONS containing 50% of recommended daily allowances for vitamin, mineral and trace elements improves micronutrient status, but micronutrient plasma levels did not reach normal levels in all participants after 4 weeks and 40% of them had still at least one micronutrient deficiency. We believe that vitamin, mineral and trace element supplements should be routinely prescribed in combination with energy and protein dense ONS in institutionalized older adults at the beginning of their nutritional care. Further studies will be needed to determine the doses and duration of micronutrient supplementation that are needed to correct micronutrient status in institutionalized older adults. The issue of correcting micronutrient deficiencies should also be addressed in older malnourished hospitalized patients and in those living at home.

## Data availability statement

The raw data supporting the conclusions of this article will be made available by the authors, without undue reservation.

## Ethics statement

The studies involving humans were approved by Comité de Protection des Personnes Ile-de-France—Paris VI. The studies were conducted in accordance with the local legislation and institutional requirements. The participants provided their written informed consent to participate in this study.

## Author contributions

Conceptualization: LC, JG, CA, OB, and AR-S. Methodology: NN, LB, CA, AR-S, and LC. Validation, formal analysis, and data curation: LB, MS, and PC-A. Investigation and project administration: JG and BD. Writing—original draft preparation: AC, AR-S, MS, and PC-A. Writing—review and editing: AC, NN, MS, PC-A, AR-S, OB, CA, BD, and LC. Supervision: AR-S and LC. Funding acquisition: LC. All authors have read and agreed to the published version of the manuscript.
