# Supplementary material for: Four-week administration of an energy and protein dense oral nutritional supplement improves micronutrient concentrations but does not completely correct deficiencies in institutionalized malnourished older adults
Source: Front Nutr. 2023 Sep 27;10:1249936. doi: 10.3389/fnut.2023.1249936 (PMC10565821; doi:10.3389/fnut.2023.1249936)
Supplement: Supplementary file 1 [file Table_1.DOCX]

Supplementary Material

Four-week administration of an energy and protein dense oral nutritional supplement improves micronutrient concentrations but does not completely correct deficiencies in institutionalized malnourished older adults.

**Manuel Sanchez^1,2*^, Pauline Courtois-Amiot^1^, Audrey Capdepon^3,4^, Nathalie Neveux^4,5^, Julien Gautry^3^, Béatrice Dorigny^3^, Ludovic Brossault^6^, Olivier Bouillanne^4,7^, Christian Aussel^4,8^, Agathe Raynaud-Simon^1,2,4^ and Luc Cynober^4,5^**

^1^ Department of Geriatrics, APHP Bichat Hospital, Paris, France

^2^ Faculty of Medicine, Paris Cité University, Paris, France

^3^ Nestlé Health Science, Issy les Moulineaux, France

^4^ URP 4466, Faculty of Pharmacy, Paris Cité University, France

^5^ Clinical Chemistry Laboratory, APHP Cochin Hospital, Paris, France

^6^ Soladis Group, Lyon, France

^7^ Department of Geriatrics, APHP Emile Roux Hospital, Limeil Brevannes, France

^8^ Clinical Chemistry Laboratory, APHP Saint-Antoine Hospital, Paris France

*** Correspondence:** Corresponding Author: [manuel.sanchez@aphp.fr](mailto:manuel.sanchez@aphp.fr)

**Supplementary Table 1:** Analytical characteristics of micronutrient assays.

|  | Ref for normal range | Aliquots | Equipment | Detection limits | Within run CV % | Between run CV % | Reference material |
| --- | --- | --- | --- | --- | --- | --- | --- |
| Magnesium [0.74-1.03 mmol/l] | Roche Data | 2 | Modular (Roche) | 0.10 mM | 0.54 | 2.8 | Liquichek Unassayed chemistry control (Biorad) |
| Selenium [0.89-1.65 µmol/l] | Cerba laboratory data | not provided by the laboratory | | | | | |
| Zinc  [11-24 µmol/l] |  |  |  |  |  |  |  |
| Vitamin A [1.5-2.7 µmol/l] | Cochin laboratory data | 2 | Summit (Dionex) | 0.3 µmol/L | 4.52 | 5.28 | Serum Control Bi level Vit A-E (Chromsystems) |
| Vitamin E [3-9 µmol/mmol] |  |  |  | 1 µmol/L | 4.49 | 4.79 |  |
| Vitamin C [5-15 mg/l] | Chromsystems Data | 2 |  | 0.4 mg/L | 2.62 | 5.32 | Plasma Control Vit C  (Chromsystems) |
| Vitamin B9 erythrocyte [260-1030 µg/l] | Roche Data | 2 | Cobas E (Roche) | 46.5 µg/L | 2.9 | 7.6 | Lyphochek Whole Blood (Biorad) |
| Vitamin B9 serum [3.6-17.0 µg/l] | Roche Data | 2 |  | 1.2 µg/L | 2.8 | 9.8 | PreciControl Varia (Roche) |
| Vitamin B12 [223-1100 ng/l] | Roche Data |  |  | 100 ng/L | 1.2 | 6.3 |  |
